# Accuracy of drug advertisements in medical journals under new law regulating the marketing of pharmaceutical products in Switzerland

**DOI:** 10.1186/1472-6947-8-61

**Published:** 2008-12-31

**Authors:** Macarena Gonzalez Santiago, Heiner C Bucher, Alain J Nordmann

**Affiliations:** 1 Basel Institute for Clinical Epidemiology, University Hospital Basel, Hebelstrasse 10, CH-4031 Basel, Switzerland

## Abstract

**Background:**

New legal regulations for the marketing of pharmaceutical products were introduced in 2002 in Switzerland. We investigated whether claims in drug advertisements citing published scientific studies were justified by these studies after the introduction of these new regulations.

**Methods:**

In this cross-sectional study, two independent reviewers screened all issues of six major Swiss medical journals published in the year 2005 to identify all drug advertisements for analgesic, gastrointestinal and psychopharmacologic drugs and evaluated all drug advertisements referring to at least one publication. The pharmaceutical claim was rated as being supported, being based on a potentially biased study or not to be supported by the cited study according to pre-specified criteria. We also explored factors likely to be associated with supported advertisement claims.

**Results:**

Of 2068 advertisements 577 (28%) promoted analgesic, psychopharmacologic or gastrointestinal drugs. Among them were 323 (56%) advertisements citing at least one reference. After excluding multiple publications of the same drug advertisement and advertisements with non-informative references, there remained 29 unique advertisements with at least one reference to a scientific study. These 29 advertisements contained 78 distinct pairs of claims of analgesic, gastrointestinal and psychopharmacologic drugs and referenced studies. Thirty-seven (47%) claims were supported, 16 (21%) claims were not supported by the corresponding reference, and 25 (32%) claims were based on potentially biased evidence, with no relevant differences between drug groups. Studies with conflict of interest and studies stating industry funding were more likely to support the corresponding claim (RR 1.52, 95% CI 1.07–2.17 and RR 1.50, 95% CI 0.98–2.28) than studies without identified conflict of interest and studies without information on type of funding.

**Conclusion:**

Following the introduction of new regulations for drug advertisement in Switzerland, 53% of all assessed pharmaceutical claims published in major medical journals are not supported by the cited referenced studies or based on potentially biased study information. In light of the discrepancy between the new legislation and the endorsement of these regulations, physicians should not trust drug advertisement claims even when they seem to refer to scientific studies.

## Background

Drug advertisement is an effective tool to form physicians' perception of drug efficacy and prescription behaviour [1-4]. Advertisement claims of pharmaceutical companies have been criticized for making exaggerated claims, emphasizing relative over absolute effect measures [5], omission of adverse effects, and for use of different standards for promoting drugs in resource-limited countries [6].

In step with the growing importance of evidence-based medicine, we have observed the pharmaceutical industry in Switzerland increasingly including bibliographical references in drug advertisements to give apparent support to promotional claims. The accuracy of drug advertisements in medical journals and direct-to consumer advertisements in relation to cited scientific material has been addressed by various investigations and inaccurate or misleading claims in drug advertisements have been identified [7-16].

On January 1^st ^2002, Switzerland introduced the new Federal Act on Therapeutic Products that defines new regulations for the marketing of pharmaceutical products [17]. The law requires that advertisements claims "have to be accurate, balanced and supported by evidence". Marketing claims must "reflect current scientific knowledge" and "only studies conducted according to good clinical practice" may be cited. Claims in advertisements "must be quoted accurately, completely and need to be exactly referenced" [17].

In this study, we evaluate the accuracy of advertisement claims by the pharmaceutical industry that have been published in the six most widespread medical journals in Switzerland 3 years following the introduction of the new Federal Act on Therapeutic Products. In addition, we evaluated whether claims were based on potentially biased studies. We also explored factors likely to be associated with supported advertisement claims such as claims referring to studies with industry funding, claims referring to studies with potential conflict of interest, and claims referring to studies with double blind study design.

We assumed that this time period would be sufficient to allow for the adaptation to the new requirements. We focused our research on claims in pharmaceutical advertisements for analgesic, gastrointestinal and psychopharmacologic drugs for three reasons. They belong to the most frequently prescribed drugs in primary care, no previous study has addressed the adequacy of advertisement claims for these drug categories and because we expected referenced studies relating to these drugs report functional or quality of life related endpoints that are of immediate importance to patients.

## Methods

In this cross-sectional study, we screened all issues of six major Swiss medical journals [18-23], including the official weekly organ of the Swiss Medical Association (FMH) [20] published in the year 2005 to identify all drug advertisements for analgesic, gastrointestinal and psychopharmacologic drugs (Table 1). Five of these journals target general practitioners and internists in Switzerland.

**Table 1:** Drug advertisement in 6 major Swiss medical journals in the year 2005

Journal name	Swiss Medical Weekly[18]	Schweizerische Ärztezeitung[20]	Swiss Medical Forum[19]	Therapeutische Umschau[22]	Therapiewoche[23]	Ars Medici[21]
Issues per year (2005)	48	52	52	12	12	26
Peer reviewed	Yes	No	Yes	Yes	No	No
Listed in Medline	Yes	Yes	Yes	Yes	No	No
Type of subscription	By charge	By membership	By membership	By charge	Free of charge	Free of charge
Circulation (copies per issue)	1600	34800	34800	3000	5000	9500
Total Pages:	715	2845	1312	800	270	1152
Pages with drug advertisements	68 (8.6%)	751 (20.2%)	555 (26.8%)	249 (22.2%)	73 (19.5%)	372 (23%)
Pages by drug category	22	117	52	24	12	
- Analgesic drugs	8	78	59	14	7	50
- Psychopharmacological drugs	2	33	31	1	11	34
- Gastroenterological drugs						22

Two reviewers first independently quantified the total number of advertisements, and then the number of unique advertisements. Thereafter they independently evaluated all drug advertisements referring to at least one scientific publication according to pre-specified criteria. We abstracted from each cited study data on the type of study (systematic review or meta-analysis, randomised controlled trial, cohort study, case control study, case series, or narrative review), financial source of support, declaration of potential conflict of interest, six quality criteria for randomised controlled trials, the type of study endpoint (clinical or surrogate endpoint) and whether endpoints were specified as primary, secondary, or not pre-specified (post hoc).

Advertisements were independently categorised into the following categories of claims: efficacy, reduction in adverse effects (safety), cost (reduced costs or better cost-effectiveness ratio), and dosage/convenience (simplified dosing or ease of use). We then classified claims as supported, not supported or based on potentially biased evidence. Claims with vague or general statements that could not be verified by a referenced study were excluded from our analysis such as claims referring to the Swiss Drug Compendium, the standard reference book for drugs licensed in Switzerland [24].

A claim was rated as not being supported by the cited study, if one of the following criteria applied: false statement, absence of relation, exaggeration of efficacy, unjustified generalization or unjustified transfer to humans. False statement was defined as a statement that contradicted the findings of the cited study. An absence of relation existed if the advertising claim had no relation with the study referenced or the claim made by the advertising could not be inferred from the cited study. A study was rated as exaggerating efficacy if the statement from the advertisement mentioned a higher effect size that did not correspond with the effect size in the original study. Unjustified generalization was defined as an advertising claim that targeted patient groups other than patients evaluated in the cited study (e.g. different age or gender groups). If an effect was observed in-vitro or in animal studies only, we rated the claim as representing an unjustified transfer of results to humans.

An advertising claim was rated as being based on a potentially biased study if the cited study failed at least 3 out of the following 6 quality criteria for the design or reporting of randomised controlled trials: the trial was an open study, where neither the patients nor the physicians or outcome assessor were blinded; if studies provided insufficient information about the presence or absence of concealed treatment allocation; if the number of patients lost-to-follow-up was more than 10%; if there were no reasons for drop outs reported; if there was selective reporting of positive outcomes in a study with positive and negative outcomes; and if no intention-to-treat analysis was performed. Furthermore, claims were rated as being based on potentially biased studies if cited studies referred to observational studies or to abstracts of randomised controlled trials that had not gone through a peer-review process; if there was an obvious selection bias (i.e., claim referred to case series, cohort study or to a randomised controlled trial where patients not responding or experiencing side effects to a certain drug were already excluded in the run-in-phase of the trial); if the study was based on a post-hoc analysis; and if the cited study was a narrative review (publication bias) unless the claim could be clearly substantiated or proved wrong by the narrative review. A claim was rated as supported if none of the above applied.

Disagreement between the two reviewers concerning the nature of the pharmaceutical claim, the quality of the studies and/or the decision of whether the claim was supported, not supported or based on potentially biased evidence was resolved by consensus. Where no consensus was possible, a third independent reviewer was asked to state his opinion.

The primary outcome of interest was the rate of claims supported, not supported or based on potentially biased evidence. We a priori additionally explored factors likely to be associated with supported advertisement claims such as funding of a study through the industry, potential conflict of interest of study authors, and double blind versus open randomised trials. We assumed presence of conflict of interest when at least one of the listed authors was employed by the drug company producing the marketed drug as evident from address information of each author even though no conflict of interest was officially declared.

### Statistics

We used descriptive statistics to report proportions of advertisement claims for each of the evaluated drug groups and to present the proportions of claims as being supported, not supported or being based on potentially biased evidence. We calculated risk ratios to evaluate whether author's conflict of interest or indication of industry funding increase the likelihood of supported claims. Kappa statistics were calculated for the agreement between the reviewers as to the classification of the claims as being supported, not supported or being based on potentially biased evidence [25]. All statistical analyses were conducted using Stata 9.2 (StataCorp, College Station, Texas, USA).

## Results

Characteristics of the screened six medical journals are summarised in Table 1. On average, 20% of pages in these journals were allocated to drug advertisements (range 9% to 27%). There were a total of 2068 drug advertisements and of those 577 (28%) promoted analgesic, psychopharmacologic or gastrointestinal drugs. Among them were 323 (56%) advertisements citing at least one reference.

**Figure 1:**
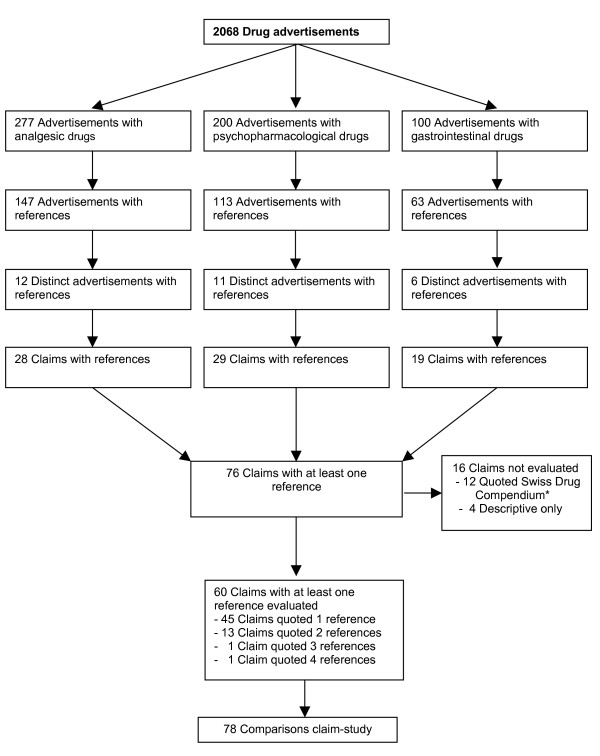
**Number of advertisements and claims with references in the study**. * The Swiss Drug Compendium is the standard reference book for drugs licensed in Switzerland [29].

After excluding multiple publications of the same drug advertisement and advertisements with non-informative references such as references referring to the Swiss Drug Compendium only, there remained 29 unique advertisements with at least one reference to a scientific study. All cited studies could be located. The 29 unique advertisements contained 76 distinct claims [Figure 1]. Sixteen of these claims did not refer to clinical studies. Most claims, (n = 66, 84%) described the qualitative effectiveness of the drug. Six claims (8%) qualitatively praised the drug's safety profile, 3 claims (4%) mentioned dosage advantages, and one claim was about a financial incentive. Among the 60 claims referring to clinical studies some claims quoted multiple references. In total, we were able to evaluate 78 distinct pairs of claims and referenced studies [Table 2]. Referenced studies comprised 51 (65%) randomised controlled trials (RCTs), 10 (13%) narrative reviews, 7 (9%) cohort studies, 4 (5%) meta-analyses or systematic reviews and 6 (7%) other types of studies (e.g. case series or laboratory studies). Sixty-seven studies (86%) addressed clinical endpoints and 11 studies (14%) reported surrogate endpoints.

**Table 2:** Non-supported claims and claims based on potentially biased evidence of drug advertisements in major Swiss medical journals in 2005

	Total	Supported claims	Not supported claims	Claims based on potentially biased evidence
Drug claims	78	37 (47%)	16 (21%)	25 (32%)
Analgesic drugs	32	14 (44%)	10 (31%)	8 (25%)
Psychopharmacologic drugs	30	17 (57%)	3 (10%)	10 (33%)
Gastrointestinal drugs	16	6 (38%)	3 (19%)	7 (43%)
				
Promotional claims				
Effectiveness	66(85%)	31 (84%)	14 (88%)	21 (84%)
Safety	6 (8%)	2 (5%)	1 (6%)	3 (12%)
Dosage/convenience	3 (4%)	1 (3%)	1 (6%)	1 (4%)
Neutral	2 (3%)	2 (5%)		
Costs	1 (1%)	1 (3%)		
				
Type of non-supported claim				
False statement			4 (25%)	
Exaggeration			4 (25%)	
Unjustified generalization			3 (19%)	
Absence of relation			3 (19%)	
Unjustified transfer to humans			2 (13%)	
				
Type of claims based on potentially biased evidence				
Abstract only				9 (36%)
Selection bias				6 (24%)
Publication bias				5 (20%)
Information of ≥ 3 quality items of RCT* missing				4 (16%)
Post hoc analysis				1 (4%)

RCT*, randomised controlled trial

Forty-one studies of the 78 claim-study pairs (53%) did not provide any information about the financial source of the study, 35 studies (45%) were industry funded, and one study each was sponsored by a non-profit organization (1%) or combined funding from a profit and a non-profit organization (1%). No study reported authors' conflict of interest, although we identified a conflict in 47 of studies (60%) where at least one of the listed authors was employed by the drug company producing the marketed drug.

### Accuracy of Pharmaceutical Claims

Thirty-seven (47%) claims were supported and 16 (21%) claims were not supported by the corresponding reference, 25 (32%) claims were rated to be based on potentially biased evidence with no relevant differences between drug groups [Table 2]. The kappa statistics for agreement of claims rated as supported, not supported and being based on potentially biased evidence were 0.77, 0.73, and 0.71, respectively. Reasons for not supported claims were false statements in 4 claims (24%), exaggerations of efficacy in 4 claims (24%), unjustified generalizations in 3 claims (19%), absence of relation in 3 claims (19%), and unjustified transfer to humans in 2 (13%) claims. Reasons for potentially biased claims were claims referring to abstracts only (n = 9, 36%), to studies with selection bias (n = 6, 24%), to studies suffering from publication bias (n = 5, 20%), to randomised trials with three or more quality criteria not being reported (n = 4, 16%), and to one study reporting a post-hoc analysis (n = 1, 4%). Examples of each category of not supported or potentially biased claims are provided in Tables 3 and 4. For example, a striking unjustified transfer to humans was a claim for Duspatalin^® ^(mebeverine) stating that Duspatalin^® ^"is effective in all cases of irritable bowel syndrome" with a reference to a study investigating the drug's effect in the bowel of pigs [26]. Another advertisement promoting Duphalac^® ^(lactulose) as having a "fast and permanent effect in constipation" referred to a study limited to evaluation of the effect of lactulose on the composition of bowel flora [27].

**Table 3:** Examples of not supported claims

**Type**	**Drug**	**Claim**	**Reference**	**Reasons for non-support**
**False statement**	Tilur^®^(Acemetacin)	As effective as the best, as safe as the	Leeb et al. *Orthopädie *33: 1032–1041, 2004	Only comparator to acemetacin was diclofenac which is neither the most effective nor the safest non-steroidal antirheumatic drug
**Exaggeration of efficacy**	Relpax^® ^(Eletriptan)	Eliminates pain	Sandrini G et al. *Neurology *59:1210–1217, 2002	At 24 hours only 24%(40 mg) and 29%(80 mg) of patients treated with eletriptan were pain-free
**Unjustified generalization**	Xefo^® ^(Lornoxicam)	Safe for heart and circulation	Tsurko V et al. *Ter Arkh *74:63–6, 2002	Only blood pressure and heart rate measured. No hard endpoints evaluated
				
**Absence of relation**	Duphalac^® ^(Laktulose)	Fast and permanent effect on constipation	Ballongue et al. *Scand J Gast *32 Suppl. 222: 41–44, 1997	Constipation not an outcome of the study.
**Unjustified transfer to humans**	Duspatalin^® ^(Mebeverin)	Effective in all cases of irritable bowel syndrome	Boisson J et al. *Chir. Digest*.16: 289–292, 1987	Animal study (pigs)

**Table 4:** Examples of claims based on potentially biased evidence

**Type**	**Drug**	**Claim**	**Reference**	**Reasons for potential bias**
**Abstract**	Pantozol^® ^(Pantoprazol)	Similar healing of reflux disease as omeprazol 20 mg	Bardnan KD et al. *Gastroenterology *116(Suppl.4II): A 118, 1999	Abstract, open study, no blinded outcome assessment
				
**Selection Bias**	Neurodol^® ^(Lidocaine)	Neurodol tissugel reduces pain and allodynia in postherpetic neuralgia	Meier T et al., *Pain *106: 151–158, 2003	Selection bias (only patients who had already successfully been treated with lidocaine for more than 1 month were included in study), very few patients
				
**Publication bias**	Lamictal^® ^(Lamotrigine)	Well tolerated	Bowden CL et al. *Drug Safety *2004; 57: 173–184	Narrative review, possible publication and selective reporting of evidence bias
				
**At least 3 quality criteria missing**	Zoloft^® ^(Sertraline)	Increases cognitive capacity	Newhouse PA et al. *J Clin Psych *2000;61:559–68	High drop out rate(>30%), no descriptions of losses to follow-up, selective reporting of positive outcomes
				
**Post hoc analysis**	Jarsin^® ^(St. John's word)	Similar efficacy as synthetic antidepressants	Brenner R et al. *Clinical Therapeutics *22: 411–419, 2000	Post-hoc analysis, only 15 patients in each group, no sample size calculation

### Association of claims referring to industry-funded studies and studies with potential conflict of interest with supported advertisement claims

Advertisement claims were more likely to be supported by referenced studies where at least one author had a conflict than studies without identified conflict of interest (RR 1.52, 95% CI 1.07–2.17). Similarly, claims were more likely to be supported by referenced studies with identified funding than studies not indicating the source of funding (RR 1.50, 95% CI 0.98–2.28).

There were 51 randomised controlled trials among the referenced studies. In 21% of these trials random allocation was concealed, 92% were double blind trials, only 6% reported blinded outcome assessment, 51% reported complete description of all losses to follow-up and withdrawals, 23% reported a lost to follow-up of less than 10%, and 75% of trials were analyzed according to the intention-to-treat principle. There was a trend that claims referring to studies with double blind design were more likely to be supported than trials with an open design (RR 1.15, 95%CI 0.94–1.41).

## Discussion

In this representative survey on the accuracy of pharmaceutical claims in medical journals in Switzerland, only 47% of all claims for analgesic, gastrointestinal and psychopharmacologic drugs were supported by the referenced study. More than half of all claims were not supported or based on potentially biased information. In our opinion, these claims are in violation of the Federal Act on Therapeutic Products regulating the marketing of medical drugs in Switzerland.

We assessed all pharmaceutical claims referring to analgesic, gastrointestinal and psychopharmacologic drugs for the year 2005 in all issues of six major medical journals in Switzerland according to predefined criteria. The agreement between the two independent reviewers with respect to the classification of claims into the three categories of being supported, not supported or based on potentially biased referenced studies was substantial, thereby increasing the credibility of our assessment [25].

Our study has a few limitations. Only a small fraction of all advertisements contained claims referring to scientific studies. Thus, our analysis is based on relatively few pairs of claims and referenced studies. Our systematic search identified 323 advertisements referring to analgesic, gastrointestinal and psychopharmacological drugs citing at least one reference. Claims with vague or general statements that could not be matched to a referenced study were excluded from our analysis. After excluding multiple publications of the same advertisement, we ended up with 78 pairs of claims and referenced studies, because advertisements of the same product were repeatedly placed in several issues of medical journals. We did not calculate kappa statistics for all classifications, but limited them to the classification of the claims as being supported, not supported or being based on biased evidence. For several reasons, we limited our assessment of the accuracy of pharmaceutical claims to drugs that are very frequently used in primary care for treatment of functional disorders that are of immanent and immediate importance to patients. The accuracy of pharmaceutical claims referring to lipid-lowering and antihypertensive drugs has already been assessed recently [6]. Furthermore, we hypothesized that referenced studies relating to analgesic, gastrointestinal and psychopharmacologic drugs may be more prone to biased interpretation than studies relating to lipid-lowering and antihypertensive drugs. Outcomes such as reduction in pain or symptom scores are harder to quantify and may not be as reliable as opposed to outcomes in cardiovascular studies such as mortality or myocardial infarction. However, in a study by Villanueva et al. assessing the accuracy of all advertisement claims for antihypertensive and lipid-lowering drugs in six Spanish medical journals [6], 44% of promotional statements were not supported by the cited reference study, only slightly less than in our study. We used a very similar methodology to rate advertisement claims like in this study from Spain, but expanded the assessment on quality aspects of referenced trials. In the study by Villanueva [6], the most common reason for not supported claims were recommendations to use the drug in a patient group other than the one assessed in the referenced study. In our survey, the most common reasons for not supported claims were false statements and exaggeration of efficacy. These differences might be explained by the type of drugs evaluated. Antihypertensive and lipid-lowering drugs are often evaluated in large scale randomised controlled trials of highly selected patients powered for clinical endpoints such as myocardial infarction and overall mortality. Therefore in the context of aggressive marketing and expansion of drug use misquotes are more likely to be related to extrapolation of effects to patient populations not included into the original trials. In contrast, studies on analgesic gastrointestinal or psychopharmacologic drugs are more often evaluated in smaller trials with endpoints such as pain and symptom relief or quality of life. These endpoints are often difficult to interpret for clinicians in the context of the clinical relevance and thus make them more prone to reports of exaggerated efficacy or false statements.

In a Finnish study assessing the quality of marketing claims medical journals published in Finland in 2002 [12], only 2% of marketing claims were rated as being supported by strong scientific evidence. The extremely low proportion of supported claims in that study, however, is explained by the fact that authors rated a high number of marketing claims with vague or emotional statements (68% of all assessed claims) as not supported by the referenced study. In contrast, we excluded claims with vague or general statements that could not be verified by a referenced study from our analysis such as purely descriptive claims referring to the Swiss Drug Compendium, the standard reference book for drugs licensed in Switzerland [24].

In a study evaluating claims of rheumatologic drug advertisements in four rheumatologic specialist journals, 74% claims were rated as being poorly supported and 8% as being misleading [28]. However, this study included all claims with references that related to data on file or official prescribing information which then were rated as poor evidence support whereas we excluded all claims with references to prescribing or on file information.

We found that studies with conflict of interest and studies stating industry funding were more likely to support the corresponding claim, although this particular information was missing in the majority of studies. Thus, in this survey active involvement of the drug manufacturer in the study seems to be associated with more accurate citation of study findings and more directive and professional marketing. However, some of these studies may still have been biased in ways we could not detect by looking at study reports alone.

Our findings demonstrate that in Switzerland many pharmaceutical advertisement claims referencing scientific information currently do not accord with the Federal Law on Therapeutic Products [17] and indicate a problematic discrepancy between current legislation and the existing passive control mechanisms for the adequacy of drug advertisements. Publishers of medical journals do not take responsibility for the content of advertisement claims and usually exert only rudimentary controls to avoid the publication of offensive, aggressive, or exaggerated advertisement claims. Swissmedic, the Swiss Agency for Therapeutic Products, is responsible to take legal action against violation of the Federal Act on Therapeutic Products. The agency must react when informed about inaccurate pharmaceutical advertisement claims, but has no system in place to actively survey the adequacy of advertisement claims of pharmaceutical companies for example by random sample checking. This is particularly problematic since drug advertisement is known to influence prescription patterns of physicians. Although physicians perceive themselves as paying little attention to drug commercials, some evidence suggests that prescription patterns of physicians may be influenced by drug advertisements [4,29-34].

Our study has important implications. Physicians should not trust pharmaceutical advertisement claims even when they are referring to scientific studies. Physicians need to be aware that the pharmaceutical industry may use drug advertisements to influence prescription patterns even when this results in distortion of scientific facts. The pharmaceutical industry should be more responsible and more meticulous in making sure that pharmaceutical claims referring to scientific studies are quoted accurately. Given the magnitude of unsupported drug claims observed in our study, Swissmedic and similar organizations in other countries should be required to take an active monitoring role in checking the accuracy of drug advertisements. Legal health entities, the pharmaceutical industry and publishers of medical journals may need to be held responsible to develop and promote minimal standards for the publication of pharmaceutical drug advertisements. Control mechanisms that may be reasonably applied like for example a random peer review process according to a standardized checklist should be adopted. Recently, a checklist for reporting an abstract of a randomized trial has been suggested as a mean to provide the necessary details and clarity in abstracts allowing readers to assess the validity and applicability of randomised trial results[35]. A similar instrument could add to create standards to improve the accuracy of information provided in drug advertisement claims.

## Conclusion

We conclude that less than half of all advertisement claims for analgesic, gastrointestinal or psychopharmacologic drugs published in Swiss medical journals were supported by the referenced studies. Physicians need to be aware that they can not blindly trust drug advertisement claims even when they seem to refer to scientific studies. In our opinion, legal actions such as administrative fines may be required to ensure systematic monitoring of the accuracy of pharmaceutical marketing claims in order to improve the accuracy of pharmaceutical advertisement claims in the future.

## Competing interests

The authors declare that they have no competing interests.

## Authors' contributions

MGS interpreted the data and helped drafting the manuscript. HCB interpreted the data and revised it critically for important intellectual content. AJN designed the study, performed the statistical analysis, and drafted the manuscript. All authors read and approved the final manuscript.

We assure that all authors included on a paper fulfil the criteria of authorship. In addition we assure that there is no one else who fulfils the criteria but has not been included as an author. AJN is the guarantor of this manuscript.

## Pre-publication history

The pre-publication history for this paper can be accessed here:


